# Demise of Another Medical Journal

**Published:** 1847-08

**Authors:** 


*Demise of another Medical Journal.*—We regret to state that the *Lancette Canadienne*, a Journal in the French language, recently established in Montreal, has been discontinued for *want of support*.

